# Cone-Beam Composite-Circling Scan and Exact Image Reconstruction for a Quasi-Short Object

**DOI:** 10.1155/2007/87319

**Published:** 2008-02-03

**Authors:** Hengyong Yu, Ge Wang

**Affiliations:** Biomedical Imaging Division, VT-WFU School of Biomedical Engineering and Science, Virginia Polytechnic Institute and State University, Blacksburg, VA 24061, USA

## Abstract

Here we propose a cone-beam composite-circling mode to solve the quasi-short object problem, which is to reconstruct a short portion of a long object from longitudinally truncated cone-beam data involving the short object. In contrast to the saddle curve cone-beam scanning, the proposed scanning mode requires that the X-ray focal spot undergoes a circular motion in a plane facing the short object, while the X-ray source is rotated in the gantry main plane. Because of the symmetry of the proposed mechanical rotations and the compatibility with the physiological conditions, this new mode has significant advantages over the saddle curve from perspectives of both engineering implementation and clinical applications. As a feasibility study, a backprojection filtration (BPF) algorithm is developed to reconstruct images from data collected along a composite-circling trajectory. The initial simulation results demonstrate the correctness of the proposed exact reconstruction method and the merits of the proposed mode.

## 1. INTRODUCTION

Since its introduction in 1973 [1], X-ray CT has revolutionized clinical imaging and become a cornerstone of radiology departments. Closely correlated to the development of X-ray CT, the research for better image quality at lower dose has been pursued for important medical applications with cardiac CT being the most challenging example. The first dynamic CT system is the dynamic spatial reconstructor (DSR) built at the Mayo Clinic in 1979 [2, 3]. In a 1991 SPIE conference, for the first time we presented a spiral cone-beam scanning mode to solve the long object problem [4, 5] (reconstruction of a long object from longitudinally truncated cone-beam data). In 1990s, single-slice spiral CT became the standard scanning mode of clinical CT [6]. In 1998, multislice spiral CT entered the market [7, 8]. With the fast evolution of the technology, helical cone-beam scanning becomes a main mode of clinical CT. Moreover, just as there have been strong needs for clinical imaging, there are equally strong demands for preclinical imaging, especially of genetically engineered mice [9–11].

To meet the biomedical needs and technical challenges, it is imperative that cone-beam CT methods and architectures must be developed in a systematic and innovative manner so that the momentum of the CT technical development as well as clinical and preclinical applications can be sustained and increased. For that purpose, our CT research has been for superior dynamic volumetric low-dose imaging capabilities. Since the long object problem has been well studied by now, we recently started working on the quasi-short object problem (reconstruction of a short portion of a long object from longitudinally truncated cone-beam data involving the short object).

Currently, the state-of-the-art cone-beam scanning for clinical cardiac imaging follows either circular or helical trajectories. The former only permits approximate cone-beam reconstruction because of the inherent data incompleteness. The latter allows theoretically exact reconstruction but due to the openness of helical scanning there is no satisfactory scheme to utilize cone-beam data collected near the two ends of the involved helical segment. Recently, saddle-curve cone-beam scanning was studied for cardiac CT [12, 13], which can be directly implemented by compositing circular and linear motions: while the X-ray source is rotated in the vertical x-y plane, it is also driven back and forth along the z-axis. Because the electromechanical needs are very challenging for converting a motor rotation to the linear oscillation and handling the acceleration of the X-ray source along the z-axis, it is rather difficult to implement directly the saddle-curve scanning mode in practice, and it has not been employed by any CT company. However, it does represent a very promising solution to the quasi-short object problem. Early this year, we invented a composite-circling scanning principle to solve the quasi-short object problem [14].

In Section 2, we will define the new scanning mode. In Section 3, we will describe a backprojection filtration- (BPF-) based exact reconstruction algorithm. In Section 4, we will present representative simulation results. In Section 5, we discuss some related issues and conclude the paper.

## 2. COMPOSITE-CIRCLING SCANNING

**Figure 1:**
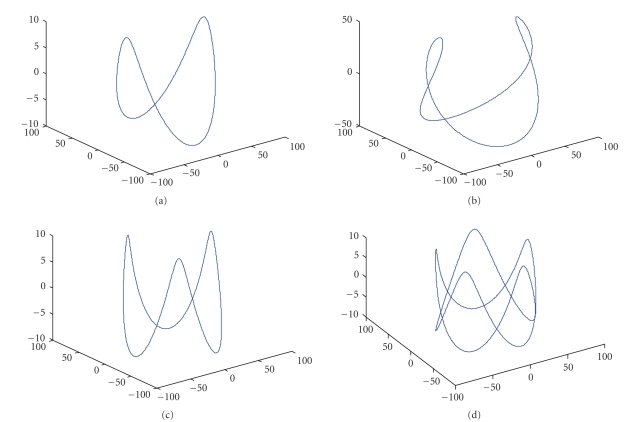
Composite-circling scanning curves with different parameter combinations. (a) m=2, $R_{1a}=R_{1b}=10$, $R_{2}=57$; (b) m=2, $R_{1a}=R_{1b}=50$, $R_{2}=57$; (c) m=3, $R_{1a}=R_{1b}=10$, $R_{2}=57$; (d) m=2.5, $R_{1a}=R_{1b}=10$, $R_{2}=57$.

When an X-ray focal spot is in a 2D (no, linear, circular, or other types) motion on the plane, or more general in a 3D motion within a neighborhood, facing a short object to be reconstructed, and the X-ray source is at the same time rotated in a transverse plane of a patient, the synthesized 3D scanning trajectory can take various forms with respect to the short object. Specifically, let $R_{1a}\geq 0$ and $R_{1b}\geq 0$ be the lengths of the two semiaxes of the scanning range in the focal spot plane facing the short object, and $R_{2}>0$ the radius of the tube scanning circle on the x-y plane, we define a family of saddle-like composite trajectory as

(1) $$\Gamma =\{\rho (s)|\begin{array}{ll} \rho_{1}(s) & =R_{2}\cos(\omega_{2}s)-R_{1b}\sin(\omega_{1}s)\sin(\omega_{2}s)\\ \rho_{2}(s) & =R_{2}\sin(\omega_{2}s)+R_{1b}\sin(\omega_{1}s)\cos(\omega_{2}s)\\ \rho_{3}(s) & =R_{1a}\cos(\omega_{1}s), \end{array}\},$$

where s∈ℝ represents time, $\omega_{1}$ and $\omega_{2}$ are the angular frequencies of the focal spot and tube rotations, respectively. When the ratio between $\omega_{1}$ and $\omega_{2}$ is an irrational number or a rational number with large numerator in its reduced form, the scanning curve covers a band of width $2R_{1a}$, allowing a uniform sampling pattern. With all the possible settings of $R_{1a}$, $R_{1b}$, $R_{2}$, $\omega_{1}$, and $\omega_{2}$, we have numerous cone-beam scanning trajectories including saddle curves and composite-circling loci that can be used to solve the quasi-short problem exactly. We are particularly interested in a rational ratio between $\omega_{1}$ and $\omega_{2}$ in this paper, which will result in a periodical scanning trajectory. Without loss of generality, we reexpress (1) as

(2) $$\Gamma =\left\{\rho (s)|\begin{array}{ll} \rho_{1}(s) & =R_{2}\cos(s)-R_{1b}\sin(ms)\sin(s)\\ \rho_{2}(s) & =R_{2}\sin(s)+R_{1b}\sin(ms)\cos(s)\\ \rho_{3}(s) & =R_{1a}\cos(ms) \end{array}\right\},$$

where m>1 is a rational number. When $R_{1b}=0$ and m=2, we obtain the standard saddle curve. When $R_{1a}=R_{1b}$, we have our proposed composite-circling trajectory. Some representative composite-circling curves are shown in Figure 1.

**Figure 2:**
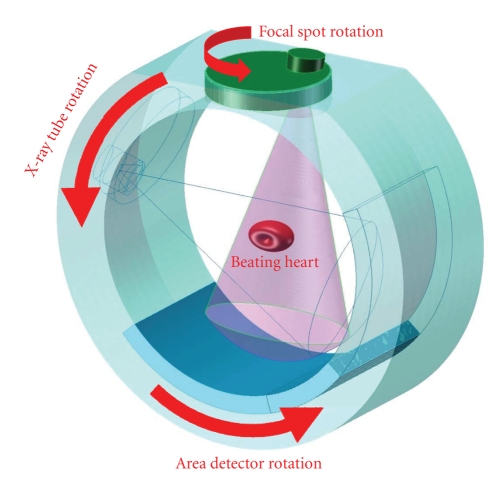
Compositing-circling scanning mode. In such a CT system, the scanning trajectory is a composition of two circular motions: while an X-ray focal spot is rotated on a plane facing a short object to be reconstructed, the X-ray source is also rotated around the object on the gantry plane. Once a projection dataset is acquired, exact or approximate reconstruction can be done in a number of ways (Copyright by Wang G, Yu HY, US Provisional Patent Application, 2007).

As mentioned in the introduction, while the saddle curve cone-beam scanning does meet the requirement for exact cone-beam cardiac CT, it imposes quite hard mechanical constraints. In contrast to the saddle curve cone-beam scanning, our proposed composite-circling requires that the X-ray focal spot undergo a circular motion in a plane facing the short object to be reconstructed, while the X-ray source is rotated in the main gantry plane (see Figure 2). Preferably, we may let the patient sit or stand straight and make the gantry plane parallel to the earth surface. Because of the symmetry of the proposed mechanical rotations and the compatibility with the physiological conditions, we believe that this approach to cone-beam CT of the short object has significant advantages over the existing cardiac CT methods and the standard saddle curve oriented systems from perspectives of both engineering implementation and clinical applications.

## 3. EXACT RECONSTRUCTION

### 3.1. Notations

Assume an object function f(r) is located at the origin of the natural coordinate system O. For any unit vector β, let us define a cone-beam projection of f(r) from a source point ρ(s) on a composite-circling trajectory by

(3) $$D_{f}(\rho (s),\beta ):=\int_{0}^{\infty }f(\rho (s)+t\beta )dt.$$

Then, we define a unit vector β as the one pointing to r from ρ(s) on the composite-circling trajectory

(4) $$\beta (r,s):=\frac{r-\rho (s)}{\left|r-\rho (s)\right|}.$$

**Figure 3:**
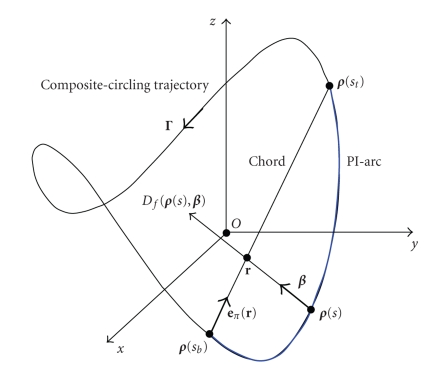
Concepts of the PI-Segment (chord) and associated PI-arc.

As shown in Figure 3, a generalized PI-line can be defined as the line through a point and across the composite-circling trajectory at two points $\rho \left(s_{b}(r)\right)$ and $\rho \left(s_{t}(r)\right)$, where $s_{b}=s_{b}\left(r\right)$ and $s_{t}=s_{t}\left(r\right)$ are the rotation angles corresponding to these two points. At the same time, the PI-segment (also referred to as a chord) is defined as the part of the generalized PI-line between $\rho \left(s_{b}(r)\right)$ and $\rho \left(s_{t}(r)\right)$, the PI-arc as the part of the scanning trajectory between $\rho \left(s_{b}(r)\right)$ and $\rho \left(s_{t}(r)\right)$, and the PI-interval as $\left(s_{b},s_{t}\right)$. All the PI-segments form a convex hull H of the composite-circling curve where the exact reconstruction is achievable according to the generalized backprojection filtration (BPF) approach [15, 16].

To perform the BPF reconstruction from data collected along a composite-circling trajectory, we define a unit vector along the chord

(5) $$e_{\pi }\left(r\right):=\frac{\rho \left(s_{t}(r)\right)-\rho \left(s_{b}(r)\right)}{\left|\rho \left(s_{t}(r)\right)-\rho \left(s_{b}(r)\right)\right|},$$

and set up a local coordinate system associated with the trajectory. Initially, we only consider the circular scanning trajectory $\overset{˜}{\Gamma }$ of the X-ray tube in the x-y plane which can be expressed as

(6) $$\overset{˜}{\Gamma }=\left\{\overset{˜}{\rho }(s)|{\overset{˜}{\rho }}_{1}(s)=R_{2}\cos(s),\;{\overset{˜}{\rho }}_{2}(s)=R_{2}\sin(s),\;{\overset{˜}{\rho }}_{3}(s)=0\right\}.$$

**Figure 4:**
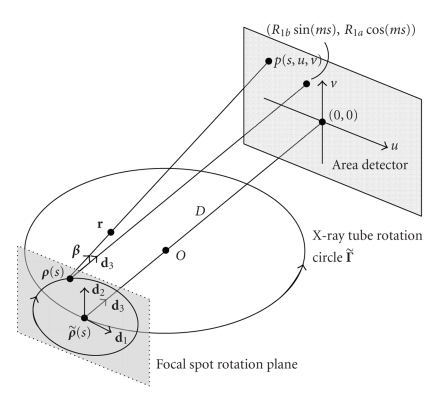
Local coordinate system with the composite-circling scanning trajectory.

For a given s, we define a local coordinate system for $\tilde{\rho }(s)$ by three orthogonal unit vectors $d_{1}:=(-\sin(s),\cos(s),0)$, $d_{2}:=(0,0,1)$, and $d_{3}:=(-\cos(s),-\sin(s),0)$ (see Figure 4). Equispatial cone-beam data are measured on a planar detector array parallel to $d_{1}$ and $d_{2}$ at a distance D from $\tilde{\rho }(s)$ with $D=R_{2}+D_{c}$, where the constant $D_{c}$ is the distance between the z-axis and the detector plane. A detector position in the array is denoted by (u,v), which are signed distances along $d_{1}$ and $d_{2}$, respectively. Let (u,v)=(0,0) correspond to the orthogonal projection of $\overset{˜}{\rho }(s)$ onto the detector array. If s is given, (u,v) are determined by β. Thus, the cone-beam projection data along a direction β from $\tilde{\rho }(s)$ can be rewritten in the planar detector coordinate system as $\tilde{p}(s,u,v):=D_{f}(\tilde{\rho }(s),\beta )$ with

(7) $$u=\frac{D\beta \cdot d_{1}}{\beta \cdot d_{3}},\;\;v=\frac{D\beta \cdot d_{2}}{\beta \cdot d_{3}}.$$

Now, let us consider the circular rotation of the focal spot at the given time s. According to our definition (2), the focal spot rotation plane is parallel to the local area detector, and the orthogonal projection of the circling focal spot position ρ(s) in the above-mentioned local area detector is $\left(R_{1b}\;\sin(ms),R_{1a}\;\cos(ms)\right)$. Thus, the cone-beam projection data along a direction β from ρ(s) can be rewritten in the same local planar detector coordinate system as $p(s,u,v):=D_{f}(\rho (s),\beta )$ with

(8) $$u=\frac{D\beta \cdot d_{1}}{\beta \cdot d_{3}}+R_{1b}\sin(ms),\;\;v=\frac{D\beta \cdot d_{2}}{\beta \cdot d_{3}}+R_{1a}\cos(ms).$$

### 3.2. Reconstruction algorithm

In 2002, an exact and efficient helical cone-beam reconstruction method was developed by Katsevich [17, 18], which is a breakthrough in the area of helical/spiral cone-beam CT. The Katsevich formula is in a filtered backprojection (FBP) format using data from a PI-arc within a slightly enlarged Tam-Danielsson window. By interchanging the order of the Hilbert filtering and backprojection, Zou and Pan proposed a backprojection filtration (BPF) formula in the standard helical scanning case [19]. This BPF formula can reconstruct an object from the data within the Tam-Danielsson window. For important biomedical applications including bolus-chasing CT angiography [20] and electron-beam CT/micro-CT [21], our group first proved the general validity of both the BPF and FBP formulae in the case of cone-beam scanning along a general smooth trajectory [15, 16, 22, 23]. Our group also formulated the generalized FBP and BPF algorithms in a unified framework [23], and applied them in the cases of generalized n-PI-window [24] and saddle curve scanning [13]. Note that our generalized BPF and FBP formulae as well as others' results [25] on general cone-beam reconstruction are valid to any smooth scanning loci, and they can be certainly applied to the reconstruction problem with the proposed composite-circling trajectory. Based on our experience with the cone-beam reconstruction from data along a saddle curve [13], the BPF algorithm is more computationally efficient than the PI-line-based FBP, and they have similar noise characteristics. Therefore, here we will use the BPF method and describe its major steps as follows.

***Step 1* (Cone-beam data differentiation)**

For every projection, compute the derivative data G(s,u,v) from the projection data p(s,u,v):

(9) $$G(s,u,v)\equiv \frac{\partial }{\partial s}D_{f}\left(\rho (s),\beta \right){|}_{\beta \;\text{fixed}}=\frac{d}{ds}p(s,u,v){|}_{\beta \;\text{fixed}}\;\;\;=(\frac{\partial }{\partial s}+\frac{\partial u}{\partial s}\frac{\partial }{\partial u}+\frac{\partial v}{\partial s}\frac{\partial }{\partial v})p(s,u,v),$$

where

(10) $$\begin{array}{ll} \frac{\partial u}{\partial s} & =\frac{{\left(u-R_{1b}\sin(ms)\right)}^{2}}{D}+D+mR_{1b}\cos(ms),\\ \frac{\partial v}{\partial s} & =\frac{\left(u-R_{1b}\sin(ms)\right)\left(v-R_{1a}\cos(ms)\right)}{D}-mR_{1a}\sin(ms). \end{array}$$

The detailed derivations of (10) are in Appendix A.

***Step 2* (Weighted backprojection)**

For every chord specified by $s_{b}$ and $s_{t}$ and for every point r on the chord, compute the weighted backprojection data

(11) $$b(r):=\int_{s_{b}(r)}^{s_{t}(r)}G(s,\overset{}{u},\overset{}{v})\frac{ds}{\left|r-\rho (s)\right|}$$

with

(12) $$\begin{array}{ll} \overset{}{u} & =\frac{D\beta (r,s)\cdot d_{1}}{\beta \cdot d_{3}}+R_{1b}\sin(ms),\\ v & =\frac{D\beta (r,s)\cdot d_{2}}{\beta \cdot d_{3}}+R_{1a}\cos(ms). \end{array}$$

***Step 3* (Inverse Hilbert filtering)**

For every chord specified by $s_{b}$ and $s_{t}$, perform the inverse Hilbert filtering along the 1D chord direction $e_{\pi }\left(r\right)$ to reconstruct f(r) from b(r). The filtering formulation is essentially the same as in our previous papers [13, 16, 24].

***Step 4* (Image rebinning)**

Rebin the reconstructed image into the natural coordinate system by determining the chord(s) for each grid point in the natural coordinate system. The rebinning scheme is the same as what we used for the saddle curve [13]. However, there are some differences in the method for determining a chord, which will be described in the next subsection.

### 3.3. Chord determination

For our composite-circling mode, we assume that $R_{1b}\leq R_{2}/(2m)$. In this case, the projection of the trajectory in the x-y plane will be a convex single curve (Appendix B). Among all the potential composite-circling trajectories, we now target the case m=2 which is similar to the popular saddle curve setting. That is, we will study how to determine a chord for a fixed point for m=2 in this subsection.

**Figure 5:**
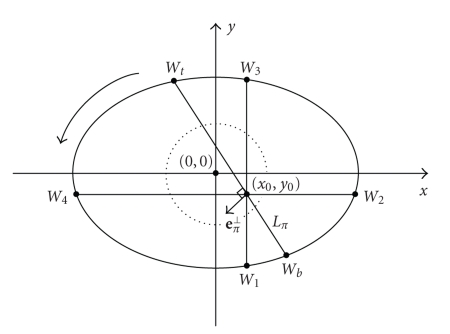
Projection of the chord and composite-circling trajectory on the x-y plane.

As shown in Figure 5, to find a chord containing the fixed point $r_{0}=(x_{0},y_{0},z_{0})$ in the convex hull H, we first consider the projection curve of the trajectory in the x-y plane. Due to the convexity of the projection curve, any line passing a point inside the curve in the x-y plane has two and only two intersections with the projection curve. Then, we consider a special plane $x=x_{0}$. In this case, there are two intersection points between the plane and the projection curve. Solving the equation $R_{2}\cos(s)-R_{1b}\sin(2s)\sin(s)=x_{0}$, that is, $R_{2}\cos(s)-2R_{1b}(1-\cos^{2}(s))\cos(s)=x_{0}$, we can obtain one and only one real root $-1\leq q_{\cos}\leq 1$ for cos⁡(s) [26], and the view angles $s_{1}=-\cos^{-1}(q_{\cos})$ and $s_{3}=-s_{1}$ that correspond to the two intersection points $W_{1}$ and $W_{3}$. On the other hand, we consider another special plane $y=y_{0}$. Solving the equation $R_{2}\sin(s)+R_{1b}\sin(2s)\cos(s)=y_{0}$, that is, $R_{2}\sin(s)+2R_{1b}(1-\sin^{2}(s))\sin(s)=y_{0}$, we have the only real root $-1\leq q_{\sin}\leq 1$ and the view angles $s_{2}=\sin^{-1}(q_{\sin})$ and $s_{4}=\pi -s_{2}$ corresponding to the two intersection points $W_{2}$ and $W_{4}$. Clearly, the above four angles satisfy $s_{1}<s_{2}<s_{3}<s_{4}$. Now, we consider a chord $L_{\pi }$ intersecting the line $L_{z}$ parallel to the z-axis through the point $\left(x_{0},y_{0},z_{0}\right)$. In the x-y plane, the projection of the line $L_{z}$ is the point $\left(x_{0},y_{0}\right)$ and the projection of $L_{\pi }$ passes through the point $\left(x_{0},y_{0}\right)$. According to the definition of a composite-circling curve, the line $\overset{\to }{W_{1}W_{3}}$ intersects $L_{z}$ at $\left(x_{0},y_{0},R_{1a}\cos(2s_{1})\right)$ while $\vec{W_{2}W_{4}}$ intersects $L_{z}$ at $\left(x_{0},y_{0},R_{1a}\cos(2s_{2})\right)$. Recall that we have assumed that $r_{0}$ is inside the convex hull H, there will be $R_{1a}\cos(2s_{1})\leq z_{0}\leq R_{1a}\cos(2s_{2})$, that is, $R_{1a}(2q_{\cos}^{2}-1)\leq z_{0}\leq R_{1a}(1-2q_{\sin}^{2})$. When the starting point $W_{b}$ of $L_{\pi }$ moves from $W_{1}$ to $W_{2}$ smoothly, the corresponding end point $W_{t}$ will change from $W_{3}$ to $W_{4}$ smoothly, and the z-coordinate of its intersection with $L_{z}$ will vary from $R_{1a}(2q_{\cos}^{2}-1)$ to $R_{1a}(1-2q_{\sin}^{2})$ continuously. Therefore, there exists at least one chord $L_{\pi }$ that intersects $L_{z}$ at $r_{0}$ and satisfies $s_{b1}\in (s_{1},s_{2})$, $s_{t1}\in (s_{3},s_{4})$. Because the composite-circling trajectory is closed, we can immediately obtain another chord corresponding to the PI-interval $\left(s_{t1},s_{b1}+2\pi \right)$. The union of the two intervals yields a 2π scan range. Similarly, we can find $s_{b2}\in (s_{2},s_{3})$ and $s_{t2}\in (s_{4},s_{1}+2\pi )$ as well as the chord intervals $\left(s_{b2},s_{t2}\right)$ and $\left(s_{t2},s_{b2}+2\pi \right)$. Hence, we can perform reconstruction at least four times for a given point inside the hull of a composite-circling trajectory. These properties are very similar to that of a saddle curve [12, 13].

Based on the above discussion, to illustrate the procedure for the chord determination, we list the following pseudocodes for numerically finding the chord corresponding to the PI-interval $\left(s_{b1},s_{t1}\right)$:

(S1) set $s_{b\min}=s_{1}$, $s_{b\max}=s_{2}$;
(S2) set $s_{b1}=(s_{b\max}+s_{b\min})/2$ and find $s_{t1}\in (s_{3},s_{4})$ so that $\vec{\rho (s_{b1})\rho (s_{t1})}$ intersects $L_{z}$:
  (S2.1) compute the unit direction $e_{\pi }^{⊥}$ in the x-y plane (see Figure 5);
  (S2.2) set $s_{t\min}=s_{3}$, $s_{t\max}=s_{4}$, and $s_{t1}=(s_{t\max}+s_{t\min})/2$;
  (S2.3) compute the projection $\delta =\left(\rho (s_{t1})-r_{0}\right)\cdot e_{\pi }^{⊥}$;
  (S2.4) if δ=0 stop, else go to (S2.2) and set $s_{t\max}=s_{t1}$ if δ>0, and set $s_{t\min}=s_{t1}$ if δ<0;
(S3) compute z′ of the intersection point between $\overset{\to }{\rho (s_{b1})\rho (s_{t1})}$ and $L_{z}$;
(S4) if $z\prime =z_{0}$ stop, else go to (S2) and set $s_{b\max}=s_{b1}$ if $z\prime >z_{0}$ and set $s_{b\min}=s_{b1}$ $z\prime <z_{0}$.

Note that $e_{\pi }^{⊥}$ in S2.1 is the direction perpendicular to $\vec{\rho (s_{b1})\rho (s_{t1})}$ and at the left side of $\vec{\rho (s_{b1})\rho (s_{t1})}$. Given the fact that implementation details of the above-described BPF method and chord determination scheme are similar to what we published in our previous papers [13, 16, 24, 27], we will not elaborate them further.

## 4. SIMULATION RESULTS

To verify the correctness of the exact reconstruction method and demonstrate the merits of the composite-circling scanning mode, we implemented the reconstruction algorithm developed in Section 3 in MatLab on a PC (2.0 Gagabyte memory, 2.8 GHz CPU), with all the computationally intensive parts coded in C. A composite-circling trajectory was made with $R_{1a}=R_{1b}=10$ cm, $R_{2}=57$ cm, and m=2.0, which is consistent with the specifications of available commercial CT scanners and satisfies the requirements for the exact reconstruction of a quasi-short object, such as the head and heart. In our simulation, the well-known 3D Shepp-Logan head phantom [28] was used. The phantom was contained in a spherical region of radius 10 cm. We also assumed a virtual plane detector and set the distance from the detector array to the z-axis ($D_{c}$) to zero. The detector array contained 523×732 detector elements with each covering 0.391×0.391 $\text{m}{\text{m}}^{2}$. When the X-ray source was moved along a turn of the composite-circling trajectory, 1200 cone-beam projections were equiangularly acquired.

**Figure 6:**
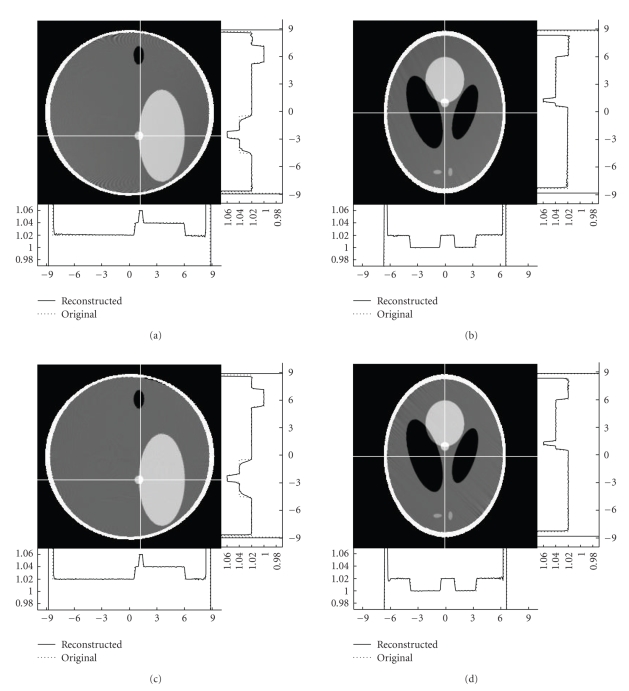
Reconstructed slices of the 3D Shepp-Logan phantom in the natural coordinate system with the display window [1, 1.05]. The top slices were reconstructed from noise-free data collected along the proposed composite-circling trajectory while the bottom ones were from a saddle curve [13]. The left and right slices were cut at X=0 cm and Z=−2.5 cm, respectively. The two profiles were plotted along the white lines in each slice.

Similar to what we did for the reconstruction in the saddle curve case, 258 starting points $s_{b}$ were first uniformly selected from the interval [−0.4492π,−0.0208π]. From each $\rho (s_{b})$, 545 chords were made with the end-point parameter $s_{t}$ uniformly in the interval $\left[s_{b}+0.8883\pi ,s_{b}+1.1150\pi \right]$. Furthermore, each chord contained 432 sampling points over a length 28.8 cm. Finally, the reconstructed images were rebinned into a 256×256×256 matrix in the natural coordinate system. Beside, our method was also evaluated with noisy datasets. We assumed that $N_{0}$ photons were emitted by the X-ray source but only N photons arrived at the detector element after being attenuated in the object, obeying a Poisson distribution. The noise standard deviations in the reconstructed images were about $3.18\times 10^{-3}$ and $10.05\times 10^{-3}$ for $N_{0}=10^{6}$ and $10^{5}$, respectively. Figures 6 and 7 illustrate some typical image slices reconstructed from noise-free and noisy datasets collected along our composite-circling trajectory, as well as the counterparts from a saddle curve [13]. While the composite-circling scanning is easier than a saddle curve in engineering implementation, there is no evident difference between the images reconstructed from the data collected along a composite-circling and a saddle curve because of their exactness. We remark that the stripe artifacts in Figure 6 were introduced by the interpolation involving phantom edges. This type of artifacts disappeared when we used a modified differentiable Shepp-Logan head phantom [29].

## 5. DISCUSSIONS AND CONCLUSIONS

To solve the quasi-short object problem, we have proposed a family of saddle-like scanning trajectories but we have only numerically evaluated the composite-circling mode with m=2. This does not mean that the case m=2 of the composite-circling mode is the optimal. We are actively working to investigate the properties of the saddle-like curves, and optimize the parameters and protocols.

Although the generalized BPF method has been developed for exact image reconstruction from data collected along a composite-circling trajectory, the method is not efficient because of its shift-variant property. Recently, Katsevich announced an important progress towards exact and efficient general cone-beam reconstruction for two classes of scanning loci [30]. The first class covers smooth and of positive curvature and torsion. The second type covers generalizes circle-plus curves [31]. Inspired by his finding, we tend to believe that there exists an exact and efficient algorithm for exact cone-beam composite-circling reconstruction. We are working hard to develop such an algorithm.

We acknowledge that for cone-beam composite-circling, we would need to rotate an X-ray tube in a plane facing a short object or have a rotating focal spot in the tube, which is not a straightforward task. However, the situation with saddle curve cone-beam scanning is even more difficult, since an X-ray tube or focal spot must be moved back and forth rapidly along the z-axis for a high longitudinal sampling rate. Given the paramount importance of exact cone-beam cardiac CT and the continued rapid development of the source and detector technology, our objective to solve the quasi-short object problem optimally with saddle-like cone-beam scanning curves is well justified. Even if neither cone-beam saddle curve scanning nor composite-circling will be implemented in the near future, the use of a fixed focal spot in a rotating X-ray tube will be likely modified or replaced soon with the use of distributed sources. We believe that in the next decade, advances in distributed and other types of X-ray sources will define a new revolution in CT, which is the hardware foundation entirely consistent with our ongoing research on cone-beam saddle-like curve-based reconstruction algorithms. Therefore, saddle-like curves, including saddle and composite-circling trajectories but not limited to them, will become increasingly important for cardiac cone-beam CT research and applications.

Regarding the engineering implementation of our composite-scanning mode, we recognize that the collimation problem must be effectively addressed [14]. Because the X-ray source, detector array, and collimators are mounted on the same data acquisition system (DAS), we can omit the rotation of the whole DAS. That is, the focal spot is circularly rotated in the plane parallel to the patient motion direction, and we need to have a collimation design to reject most of scattered photons for any focal spot position. During the scan, we can adjust the direction and position of the detector array and associated collimators to keep the line connecting the detector array center and the focal spot perpendicular to the detector plane and make all the collimators focus on the focal spot all the time. This can be mechanically done, synchronized by the rotation of the focal spot. In this case, the focal spot rotation plane and the detector plane are not parallel in general. Other designs for the same purpose are possible in the same spirit of this invention. Furthermore, our approach can also be adapted for inverse geometry based cone-beam CT [14].

In conclusion, we have developed a novel composite-circling mode and method for solving the quasi-short object problem exactly, which has better mechanical rotation stability and physiological compatibility than saddle curve scanning. Our generalized BPF method has been evaluated that reconstructs images from cone-beam data collected along a composite-circling trajectory for the case m=2. The simulation results have demonstrated the correctness and merits of the proposed composite-circling mode and exact BPF reconstruction algorithm.

## Figures and Tables

**Figure fig7:**
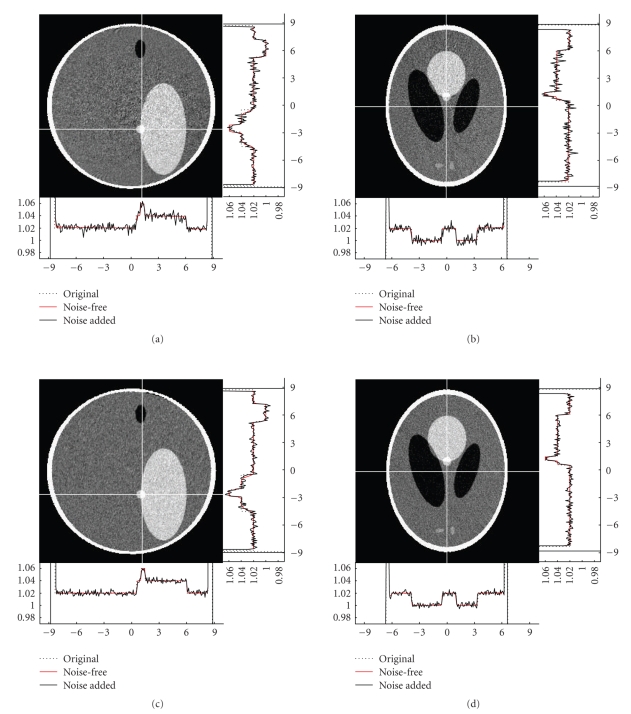
Same as Figure 6 but from noisy data with $N_{0}=10^{6}$.

## APPENDICES

### A. DERIVATIONS OF FORMULAE (10)

For a given unit direction β, its projection position in the local coordinate system can be expressed as

(A.1) $$u=\frac{D\beta \cdot d_{1}}{\beta \cdot d_{3}}+R_{1b}\sin(ms),\;\;v=\frac{D\beta \cdot d_{2}}{\beta \cdot d_{3}}+R_{1a}\cos(ms).$$

Hence, we have

(A.2) $$\begin{array}{cc} \frac{\partial u}{\partial s} & ={\left(\frac{D\beta \cdot d_{1}}{\beta \cdot d_{3}}\right)}^{\prime }=\frac{D\beta \cdot d_{1}^{\prime }}{\beta \cdot d_{3}}-\frac{D\beta \cdot d_{1}\beta \cdot d_{3}^{\prime }}{{\left(\beta \cdot d_{3}\right)}^{2}}+mR_{1b}\cos(ms),\\ \frac{\partial v}{\partial s} & ={\left(\frac{D\beta \cdot d_{2}}{\beta \cdot d_{3}}\right)}^{\prime }=\frac{D\beta \cdot d_{2}^{\prime }}{\beta \cdot d_{3}}-\frac{D\beta \cdot d_{2}\beta \cdot d_{3}^{\prime }}{{\left(\beta \cdot d_{3}\right)}^{2}}-mR_{1a}\sin(ms). \end{array}$$

Since $d_{1}^{\prime }=d_{3},\;d_{2}^{\prime }=0$ and $d_{3}^{\prime }=-d_{1}$, we obtain

(A.3) $$\begin{array}{ll} \frac{\partial u}{\partial s} & =\frac{D\beta \cdot d_{3}}{\beta \cdot d_{3}}+\frac{D{\left(\beta \cdot d_{1}\right)}^{2}}{{\left(\beta \cdot d_{3}\right)}^{2}}+mR_{1b}\cos(ms),\\ \frac{\partial v}{\partial s} & =\frac{D\beta \cdot d_{2}\beta \cdot d_{1}}{{\left(\beta \cdot d_{3}\right)}^{2}}-mR_{1a}\sin(ms). \end{array}$$

By  (A.1), it follows readily that

(A.4) $$\begin{array}{ll} \frac{\partial u}{\partial s} & =\frac{{\left(u-R_{1b}\sin(ms)\right)}^{2}}{D}+D+mR_{1b}\cos(ms),\\ \frac{\partial v}{\partial s} & =\frac{\left(u-R_{1b}\sin(ms)\right)\left(v-R_{1a}\cos(ms)\right)}{D}-mR_{1a}\sin(ms). \end{array}$$

### B. PROOF OF THE CONVEX PROJECTION CONDITION *R*_1b_ ≤ *R*_2_ */(2m)*

The projection of our composite-circling trajectory on the x-y plane can be expressed as

(B.1) $$\begin{array}{ll} & P_{\Gamma }=\{\rho (s)|\rho_{1}(s)=R_{2}\cos(s)-R_{1b}\sin(ms)\sin(s),\\ & \;\;\;\;\;\rho_{2}(s)=R_{2}\sin(s)+R_{1b}\sin(ms)\cos(s)\}. \end{array}$$

According to Liu and Traas (Lemma 2.7), a single close $C^{2}$-continuous curve is globally convex if and only if the curvature at every point on the curve is nonpositive [32]. Hence, it is required that $\rho^{\prime }(s)\times \rho^{\prime \prime }(s)\geq 0$ for any s∈ℝ. Since

(B.2) $$\begin{array}{ll} \rho_{1}^{\prime }(s) & =-R_{2}\sin(s)-R_{1b}\sin(ms)\cos(s)\\ & \;-mR_{1b}\cos(ms)\sin(s),\\ \rho_{2}^{\prime }(s) & =R_{2}\cos(s)-R_{1b}\sin(ms)\sin(s)\\ & \;+mR_{1b}\cos(ms)\cos(s),\\ \rho_{1}^{\prime \prime }(s) & =-R_{2}\cos(s)+R_{1b}(m^{2}+1)\sin(ms)\sin(s)\\ & \;-2mR_{1b}\cos(ms)\cos(s),\\ \rho_{2}^{\prime \prime }(s) & =-R_{2}\sin(s)-R_{1b}(m^{2}+1)\sin(ms)\cos(s)\\ & \;-2mR_{1b}\cos(ms)\sin(s), \end{array}$$

we have

(B.3) $$\begin{array}{ll} \rho^{\prime }(s)\times \rho^{\prime \prime }(s) & =\rho_{1}^{\prime }(s)\rho_{2}^{\prime \prime }(s)-\rho_{1}^{\prime \prime }(s)\rho_{2}^{\prime }(s)\\ & =(R_{2}\sin(s)+R_{1b}\sin(ms)\cos(s)\\ & \;\;+mR_{1b}\cos(ms)\sin(s))\\ & \;\times (R_{2}\sin(s)+R_{1b}(m^{2}+1)\sin(ms)\cos(s)\\ & \;\;\;+2mR_{1b}\cos(ms)\sin(s))\\ & \;+(R_{2}\cos(s)-R_{1b}(m^{2}+1)\sin(ms)\sin(s)\\ & \;\;\;+2mR_{1b}\cos(ms)\cos(s))\\ & \;\times (R_{2}\cos(s)-R_{1b}\sin(ms)\sin(s)\\ & \;\;\;+mR_{1b}\cos(ms)\cos(s))\\ & =(m^{2}-1)R_{1b}^{2}\cos^{2}(ms)+3mR_{2}R_{1b}\cos(ms)\\ & \;+(m^{2}+1)R_{1b}^{2}+R_{2}^{2}. \end{array}$$

Letting $z=tg^{2}(ms/2)$, we arrive at

(B.4) $$\begin{array}{ll} & \rho^{\prime }(s)\times \rho^{\prime \prime }(s)\geq 0\\ & \;\Leftrightarrow (m^{2}-1)R_{1b}^{2}{\left(\frac{1-z}{1+z}\right)}^{2}+3mR_{2}R_{1b}\left(\frac{1-z}{1+z}\right)\\ & \;\;+(m^{2}+1)R_{1b}^{2}+R_{2}^{2}\geq 0\\ & \;\Leftrightarrow \left(R_{2}^{2}+2m^{2}R_{1b}^{2}-3mR_{2}R_{1b}\right)z^{2}+2\left(R_{2}^{2}+2R_{1b}^{2}\right)z\\ & \;\;+\left(R_{2}^{2}+2m^{2}R_{1b}^{2}+3mR_{2}R_{1b}\right)\geq 0, \end{array}$$

where the relationship cos⁡(ms)=(1−z)/(1+z) has been used. Given that $R_{2}>0$, $R_{1b}\geq 0$, $2\left(R_{2}^{2}+2R_{1b}^{2}\right)>0$, and $\left(R_{2}^{2}+2m^{2}R_{1b}^{2}+3mR_{2}R_{1b}\right)>0$, we obtain the following necessary and sufficient condition for $\rho^{\prime }(s)\times \rho^{\prime \prime }(s)\geq 0$ at any s∈ℝ:

(B.5) $$R_{2}^{2}+2m^{2}R_{1b}^{2}-3mR_{2}R_{1b}\geq 0,$$

which implies that $R_{1b}\leq R_{2}/(2m)$ or $R_{1b}\geq R_{2}/m$. When $R_{1b}\geq R_{2}/m$,  the curve $P_{\Gamma }$ becomes a complex curve (not single), and this case should be excluded. Hence $R_{1b}\leq R_{2}/(2m)$ is the necessary and sufficient condition for the convex projection of the composite-circling trajectory on the x-y plane.
